# The COVID-19 Infection in Italy: A Statistical Study of an Abnormally Severe Disease

**DOI:** 10.3390/jcm9051564

**Published:** 2020-05-21

**Authors:** Giuseppe De Natale, Valerio Ricciardi, Gabriele De Luca, Dario De Natale, Giovanni Di Meglio, Antonio Ferragamo, Vito Marchitelli, Andrea Piccolo, Antonio Scala, Renato Somma, Emanuele Spina, Claudia Troise

**Affiliations:** 1 INGV, Osservatorio Vesuviano, 80124 Naples, Italy; renato.somma@ingv.it (R.S.); claudia.troise@ingv.it (C.T.); 2 CNR-INO, 80078 Pozzuoli, Italy; 3 Dipartimento di Medicina Sperimentale, Università della Campania ‘L.Vanvitelli’, 80138 Naples, Italy; valerio.ricciardi@unicampania.it; 4 Istituto Nazionale di Fisica Nucleare—Sezione di Napoli, 80126 Naples, Italy; 5 Department of Physics, University of Zurich, 8057 Zurich, Switzerland; deluca@physik.uzh.ch; 6 CoronaVerus, 80136 Naples, Italy; dario.denatale@gmail.com (D.D.N.); gds.dimeglio@gmail.com (G.D.M.); ferragamoantonio@gmail.com (A.F.); andreapiccolo.na@gmail.com (A.P.); 7 Department of Mobility, Public Works, Ecology, Env, Puglia Region Government, 70100 Bari, Italy; vitomarchitelli1@gmail.com; 8 Department of Physics ”Ettore Pancini”, Università degli Studi di Napoli “Federico II”, 80126 Naples, Italy; antonio.scala@unina.it; 9 CNR-IRISS, 80134 Naples, Italy; 10 Department of Neurosciences, Reproductive and Odontostomatology, Università degli Studi di Napoli “Federico II”, 80131 Naples, Italy; emaspina.es@gmail.com

**Keywords:** COVID-19, epidemic in Italy, statistical forecast

## Abstract

We statistically investigate the Coronavirus Disease 19 (COVID-19) pandemic, which became particularly invasive in Italy in March 2020. We show that the high apparent lethality or case fatality ratio (CFR) observed in Italy, as compared with other countries, is likely biased by a strong underestimation of the number of infection cases. To give a more realistic estimate of the lethality of COVID-19, we use the actual (March 2020) estimates of the infection fatality ratio (IFR) of the pandemic based on the minimum observed CFR and analyze data obtained from the Diamond Princess cruise ship, a good representation of a “laboratory” case-study from an isolated system in which all the people have been tested. From such analyses, we derive more realistic estimates of the real extent of the infection as well as more accurate indicators of how fast the infection propagates. We then isolate the dominant factors causing the abnormal severity of the disease in Italy. Finally, we use the death count—the only data estimated to be reliable enough—to predict the total number of people infected and the interval of time when the infection in Italy could end.

## 1. Introduction

The novel viral infection of COVID-19, recently declared a pandemic by the WHO, represents one of the most serious global emergencies of the twenty-first century. It has the potential to destroy social orders and economies and to transform our lifestyles in the near future. Since the first appearance of this new Coronavirus (SARS-CoV-2), the COVID-19 infection has been viewed in a range of ways; these vary from a perception of it as a disease no more serious than a seasonal flu to that of a highly severe and troubling threat [1]. The epidemic was first detected in the city of Wuhan, China at the end of December 2019. After a period of inaction and data suppression, the government of China showed serious concern and implemented very severe measures in the Hubei province, the centre of the epidemic, to contain the spread of the infection. Approximately 45 days after the first detection (mid-February 2020), the epidemics started to seriously affect several other countries (South Korea among the first, as it borders China). Since the end of February, infections flared up in Italy and Iran for less clear reasons and, since mid-March 2020, the epidemic has spread throughout Europe, in the USA, and many other countries [1].

Here, we focus on the COVID-19 epidemic in Italy and the features that distinguish its evolution from that observed in other countries. Though its exact parameters are uncertain, the Italian epidemic appears particularly aggressive both in terms of spread rate and lethality. In Italy, the infection is mainly focused in the Lombardia Region and the area around the Po river. The most affected regions are Lombardia, Emilia-Romagna, and Veneto; these also represent the richest and the most economically productive areas of Italy. In Italy the infection grew very quickly, surpassing South Korea and then China in the number of infected people as early as 30 March 2020 with a total of 101,739 total infections. Moreover, it showed an average case fatality ratio (CFR) over 11%. This is well above any other country and more than double that of the Hubei Region in China—where the new virus first appeared and where CFR was significantly higher than in other parts of the China and higher than other countries in the western world. 

In this paper, we present statistical analyses of data associated with the Italian COVID-19 epidemic. The aim is to estimate a possible slowdown of the infection rate and also to verify our statistical predictions in relation with the severe containment measures taken by the Italian Government. We additionally discuss alternative explanations for the very high CFR observed. To determine the true lethality or the infection fatality ratio (IFR) of the epidemic, we use the isolated, well-calibrated test case represented by the infection spread on the Diamond Princess cruise ship. Lethality (IFR) estimates obtained within the Diamond Princess are “unbiased” values not affected by underestimation of the number of infected people; IFR values computed by the University of Oxford [2] are also checked. Using various IFR estimations, we predict a much larger number of infected people in Italy than the official one that suggests that the CFR highly overestimates true lethality. We also discuss the validity of alternative hypotheses often claimed to explain the high impact of COVID-19 in Italy that are based on the influence of an older average age of the population, high antibiotic resistance, high number of smokers, and pollution in the Po plain (e.g., [2]). Finally, we identify the best data set to analyse the statistical evolution of the epidemic in Italy, comparing it with its prototypical behaviour obtained from the China dataset, to try to forecast the saturation time of the infection.

## 2. Materials and Methods

Data analysis and image processing were performed using programs written by the authors using MATLAB software (ver. 2020, Mathworks, Natick, MA, USA) and Python language [3] as interpreters. The non-linear regressions were performed using the Python module scipy.optimize.curve_fit with lm method, that implements a Levenberg-Marquardt algorithm. The results were benchmarked with the analogous outcomes of lsqcurvefit function of MATLAB that implements a trust-reflective-region algorithm. To perform the AIC test for model selection the authors implemented the algorithm [4] within the MATLAB environment.

## 3. COVID-19 in Italy

The COVID-19 epidemic in Italy presents some peculiarities that make it intriguing to analyse and understand. Initially, it was thought that two independent foci started in the towns of Codogno (15,962 inhabitants, Lodi province, Lombardia Region) and Vo’ Euganeo (3416 inhabitants, Padua province, Veneto Region), but it is now generally understood that the virus began circulating earlier throughout the entire north of Italy [5]. The epidemic rapidly expanded across Italy but particularly targeted the Po Valley in Lombardia, Veneto, and Emilia-Romagna. These are the richest regions in Italy through their industries, agriculture, and international commerce. The Lombardia and the Po Valley, the hardest hit by the epidemic, are also the most polluted areas not only in Italy but also in the whole of Europe by the fine air particulate matter (PM10, PM2.5) and ozone [6,7].

**Figure 1:**
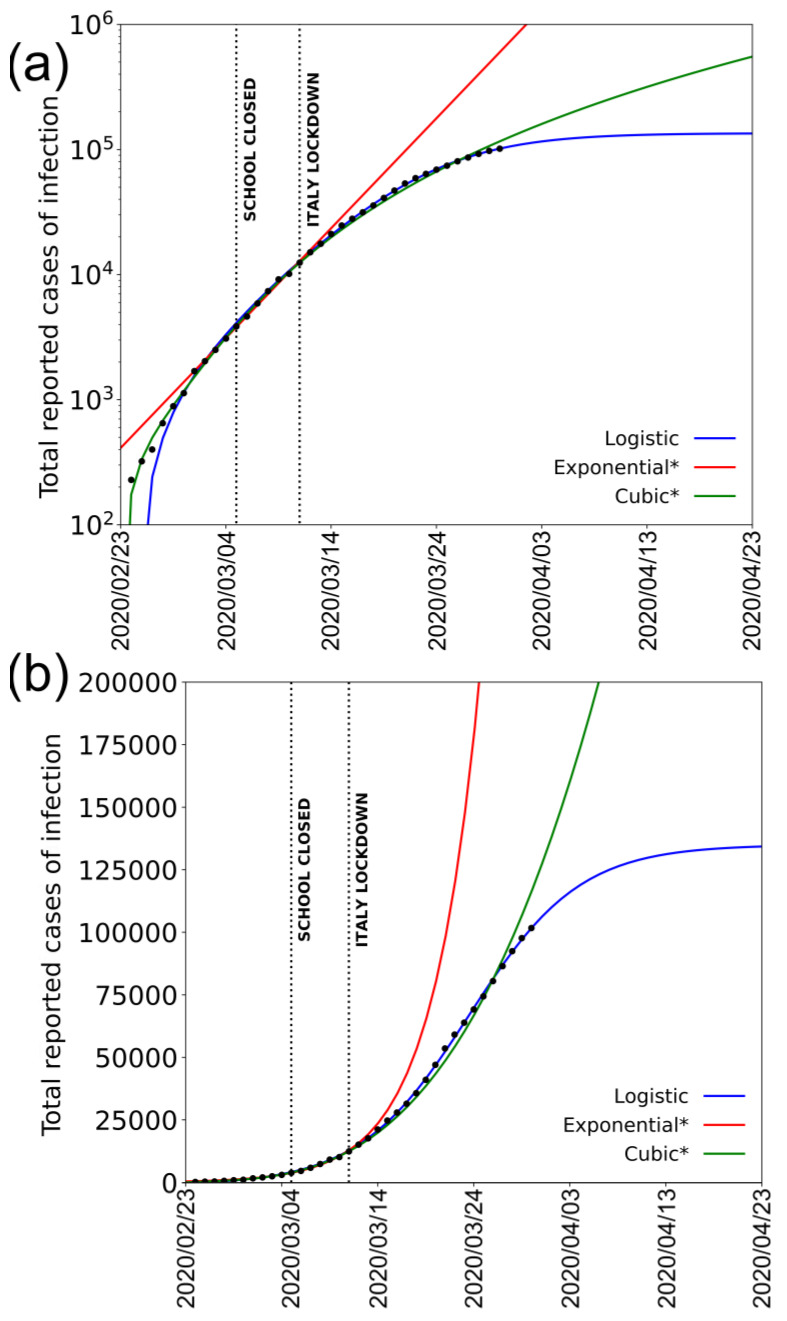
Total COVID-19 cases reported in Italy from 24 February to 30 March 2020 according to Protezione Civile (black dots) with logistic (blue solid line), exponential (red solid line), and cubic (green solid line) infection rates. Dotted black vertical lines mark the dates of Italian school lockdown and the nationwide total lockdown; the asterisk indicates that the exponential and the cubic fits are based on data until 12 March: score from Akaike Information Criterion (AIC) test (not reported) on logistic, cubic, and exponential fits shows higher reliability of the first two after this date and for the logistic against the cubic after 25 March 2020. (**a**) Fits obtained from the data in semi-logarithmic scale; (**b**) same data and fits shown in linear scale. Fit parameters: Logistic $(y=K\frac{1+me^{xr}}{1+ne^{xr}};K=\left(135\pm 2\right)\cdot 10^{3},\;m=-1.4\pm 0.2,\;n=170\pm 9,\;r=0.174\pm 0.003);$ Exponential $(y=Ae^{Bx};A=410\pm 30,\;B=0.2\pm 0.01;Cubic=a+bx+cx^{2}+dx^{3};a=-40\pm 20,\;b=36\pm 8,\;c=-6.7\pm 0.9,\;d=0.44\pm 0.03).$

The onset and the evolution of COVID-19 infection in Italy, and more specifically in Lombardia Region, was anomalous and unusually lethal with respect to all other countries, including China where the infection began. Although the media (and also many specialists interviewed by media) highlight the velocity of infection in Italy as “exponential”, the number of infected people has never followed an exponential distribution burring in the early days of the epidemic. Figure 1a shows the number of recorded infections as a function of time (in days since 24 February 2020) in a semi-logarithmic scale.

It is evident that the distribution is markedly different from a straight line, typical of an exponential distribution in such a scale, and it is fairly well fitted by a cubic polynomial, which is much slower than an exponential growth rate. We use Akaike Information Criterion (AIC) test to qualitatively weight the goodness-of-fit for the different statistical models (AIC test; [4]). The cubic polynomial fit and the exponential one present in the figure both use data referred to 18 days starting on the date of acknowledgement of the first cases (from 24 February to 12 March 2020); starting from that date, the cubic fit, according to AIC test, scores better than the exponential one. In the figure, we also show the fit obtained from a logistic function for comparison; logistic fit scores become better than the exponential one from 12 March 2020 and better than the cubic one after 25 March 2020. Figure 1b shows the same quantity in a linear scale, together with the three mentioned fitting functions.

Although the number of people infected is the main parameter taken into account by the authorities and the number highlighted by media, its real value is largely uncertain and likely underestimated. Critically, that infection tally relies on the number of laboratory tests available, which is limited and miniscule compared to the percentage of the population likely to have had contact with infected individuals. Furthermore, the procedures to test people are highly variable within the different regions of Italy and have changed in the weeks following the onset of the infections; because of this inconsistency, this number is statistically very unreliable and unfit as a foundation to interpret the actual evolution of the infection.

The number of tests performed in Italy fluctuates widely but in general increased in time from about 2427 (27 February 2020) to 26,336 (21 March 2020) and decreased again to 25,180 (22 March 2020) and 17,066 (23 March 2020). It reached a peak value of 36,615 on 26 March, but then it decreased in subsequent days [8]. A very important quantity, the CFR (defined as the ratio of the number of people deceased divided by the number of total recorded infections), is extremely high in Italy at about 11%. This high value is skewed by the lethality in Lombardia, where about 50% of all the Italian infections have been recorded, with a CFR of about 16%. CFR is a generally overestimated value of the true lethality (IFR) given the likely underestimation of the real number of infection cases (i.e., including asymptomatic and pauci-symptomatic cases, which are easily overlooked by the small number of tests). IFR is the parameter that measures the percentage of deceases over the total population infected (including the generally unknown number of non recorded cases). Table 1 [2] reports the number of infections, deaths, and CFR observed in several countries in the world as of 30 March 2020. The lethality rate for different countries is highly variable, ranging from a minimum of 0.4% (i.e., Australia and Israel) to a maximum of 11.3% in Italy. 

Several countries as of 30 March, however, had lethality rates around 1–2% (Germany, USA, Austria, Portugal, Ireland, etc.). While the reported data refer to different stages of the spread of the epidemic, it is important to note that countries at a later epidemic stage than Italy are characterized by a CFR of less than 5% (for instance China, South Korea), indicating that the high value of the CFR observed in Italy is not a general characteristic of the epidemic. It appears evident, therefore, that the CFR of Italy, and even more of Lombardia Region, is unusually high when compared to other countries. This CFR is more than double thatof the CFR obtained in China, where the epidemic first appeared. We propose that, in addition to the CFR, it is important to obtain a reasonable estimation of the IFR during the epidemic spread to understand the real hazard of the disease and/or to determine the true number of infected people.

## 4. The IFR for COVID-19 and the Lethality in Italy

The problem of unbiased estimation of the IFR for COVID-19 is complicated by the complex laboratory procedures that are necessary to identify the infected cases, ultimately limiting the total number of tested individuals. Since the IFR is the ratio between the number of deaths due to the disease and the total number of infected, the CFR, which is computed on the number of “known” infected is an upper limit of IFR. Normally, CFR (and hence IFR) should be determined at the end of an epidemic, because death occurs at the end of the epidemic cycle. Usually, at the very beginning of an epidemic, the CFR can be underestimated because the individual outcome of the disease (recovery or death) has not yet occured. For COVID-19, however, it was noted in China and in South Korea that CFR was generally higher in the first phase of the infection spread. A further problem is that the number of underestimated infected cases is generally variable and could become progressively more critical as the real number of infected rapidly rises with time while the number of tests remains stationary. Mass testing in the village of Vo’ Euganeo suggested that the number of asymptomatic persons infected by the SARS-CoV-2 is at least 50% [9]. From Table 1, we observe that each country has a very variable CFR associated with the COVID-19 infection. Since the IFR is intrinsically overestimated by the CFR, presuming the virus strain is the same in every country, we can assume the minimum value of the observed CFR as the minimum upper limit for IFR. Following this procedure, the University of Oxford used one of the minimum CFR values (around 0.4%) taken from Israel, Australia, and Iceland; then, to properly account for the undetected asymptomatics, they halved it to obtain an IFR of 0.2% [2,10]. 

For the COVID-19 epidemics, there is an independent way to obtain a rather unbiased estimate of IFR using the only “laboratory-like” case study: the Diamond Princess cruise ship anchored in the port of Yokohama from 4 February to 2 March. All 3711 passengers and crew were tested, and 712 (19.2%) persons had positive test results. Of these, 331 (46.5%) were asymptomatic at the time of testing, a similar value to the one obtained in Vo’ Euganeo. Among 381 symptomatic patients, 37 (9.7%) required intensive care, and 9 died [10,11]. Because the Diamond Princess is a perfectly isolated case in which the entire population was tested, the precise number of infected individuals is known, and we can crudely assume that IFR = CFR. The crude IFR computed from the Diamond Princess is then 1.3%. Moreover, CFR values even smaller than 1% in some countries (i.e., Australia, Norway, Israel) have been computed. In the light of such derived lethality range, the observed CFR (11.3%) in Italy appears very abnormal and in particular the CFR in the Lombardia Region (16.1%) is anomalous.

In the discussion that follows, we try to point out the possible explanations for such an anomalous outcome. We assume that the virus strain is effectively the same in every country despite possible genome variations. The first, most obvious way to explain such a high lethality is to hypothesize that the number of infected cases was substantially underestimated. A clear sign of this is the fact that lethality increased from roughly 2% at the beginning (20 February 2020) to 11% on 30 March 2020. During this period, the number of detected cases rose from few cases to around 100,000, whereas the daily number of laboratory tests changed from few hundreds to about 30,000.

In addition to the CFR, which is only linked to the recorded infection cases, it is important to understand the “local” IFR estimate for Italy in order to derive the real number of infected people. Lacking evidence for the existence of a different virus strain that is somehow more aggressive and lethal in Italy, we assume that the IFR is the same as in other places and a consistent trait of the overall pandemic. We could then choose between the University of Oxford estimate of IFR = 0.2% or the Diamond Princess laboratory-like estimation of IFR = 1.3%. If the extreme lethality observed is only due to the underestimation of the number of infected people, assuming a “true” IFR ranging between 0.2% and 1.3%, to correct for the Italian 11.3% CFR, we should either multiply by 56.5 or by 8.7, the official number of infected cases (101,739 on 30 March 2020); the resulting number would then range between about 885,000 and 5.7 million infected people, respectively, with the higher extimate in agreement with one provided by the Imperial College [12]. We also note that the CFR of whole Italy is very similar to the 11% rate observed in hospital case reports [13]. This can be explained by the tendency to only test hospitalized and severe cases excluding therefore mild and asymptomatic patients from the officially reported cases.

We cannot exclude, however, the possibility that the true IFR in Italy could be significantly higher than in other countries because of several concurrent reasons. For instance, the higher average age of the Italian people has often been indicated as a possible explanation for the high COVID-19 CFR. Globally, Italy is ranked second highest in its population’s average age; however, the top position (oldest population) is held by Japan, which showed a very low number of infections and CFR (see Table 1), thus this possibility alone appears unlikely. 

Another possible cause of comorbidity could be related to the high level of pollution in the Lombardia region, as it is considered the most polluted region in Europe by fine particulate (PM10, PM2.5) and ozone. As shown in several papers (e.g., [14,15]), there is a correlation between the diffusion of viruses and the pollution by fine particulate. Exposure to fine powders contributes to a higher severity in respiratory viral infections [16,17]. The incidence of fine particulate pollution could hence, in principle, be one of the reasons for the high lethality rate observed in Lombardia (and partially in Emilia-Romagna around the Po Valley). Although the ability of this kind of pollution to amplify the lethality observed for a severe pulmonary disease seems reasonable, it is very difficult to quantify. It is also very difficult to suggest that it can be significantly stronger than in other highly industrialized areas of Europe (Germany, France, Netherlands, etc.). The Italian National Institute of Health (ISS), in a recent position statement [18], specifies the association between air pollution and Covid-19 is still uncertain and not scientifically proven yet.

Other tentative explanations for the extreme lethality could be the high number of smokers in Italy and the antibiotic-resistance of Italian people. Regarding the percentage of smokers, however, Italy’s 23% smoking rate is lower than the European average of 29% [19]. The antibiotic-resistance, moreover, is also claimed to be a critical factor. Of the roughly 33,000 annual deaths in the EU from antibiotic-resistant bacteria, a disproportionately high number of approximately 10,000 occur in Italy (ISSa, 2019). As therapies to combat the SARS-CoV-2 infection involve one or more antibiotics, this issue could result in higher lethality in Italy. It is not easy to quantify this effect, however, which appears marginal in the normal seasonal flu outbreaks, during which the CFR for Italy is little different from that of other European countries [20]. Moreover, the use of antibiotics in COVID-19 cases is intended to avoid bacterial superinfection rather than prevent death; hence, even if there was a more pronounced antibiotic resistance, this would account for only a marginal number of deaths [21]. We can compare, for instance, the number of deaths directly or indirectly associated with seasonal flu (the number of indirect deaths is much more significant in this case, because it is mostly linked to bacteria super-infections) in Italy and in Germany (where COVID-19 CFR is about 1%). The average yearly number of such deaths in Italy is around 8000 [22]; in Germany, the deaths associated with the 2017–2018 flu, although very severe that year, totalled 25,000 [23]. 

Another possibility is that the healthcare system was unprepared for such an emergency. Prior to the COVID-19 epidemic, Italy (60 million people) had approximately 5090 beds in intensive care units (ICU); by comparison, Germany (82 million people) had 28,000 beds. In terms of number of ICU divided by population, Italy is ranked an ignominious 19th out of 23 European Countries. We indeed observe high pressure on the ICU system by severe and critical cases, particularly in Lombardia, where the number of ICU beds before the crisis was 900; this number increased to over 1000, but, as of 30 March 2020, over 1300 patients were hosted in ICU facilities. On 14 March, the government of Lombardia declared that almost all available ICU beds were filled and no remaining facilities existed. Another indication that something went wrong during the first phase of management of the infection is the very high number (6414) of infected medical staff in the Lombardia hospitals [24]. The hospitals themselves may have been the most effective transmitters of the virus in the first phase of rapidly increasing infections in Lombardia, as observed also in Wuhan [25]. 

## 5. Possible Forecast of Future Behaviour of Infection

In order to forecast the evolution and the end of the COVID-19 epidemic in Italy, we could, in principle, use three kinds of data. The most obvious would be the daily infection rate data. However, such data are particularly unreliable because they are highly dependent on the changing number of tests available on a daily basis. They are generally very variable and inconsistent both within and between regions as well as over time. Since, as we noted, the real number of infections is likely much higher (by orders of magnitude) than the sampled number, the inhomogeneous sampling can strongly condition the number of infections and make them not useful for a statistical study. Another possible indicator of the epidemic evolution could be the number of people in an ICU. Contrary to the number of people infected, the number of people in an ICU should be objective because only those with serious respiratory problems will be hospitalized with that level of care. However, in this phase of the epidemic crisis, this number has two major problems; the first is that the ICU capacity was overwhelmed, particularly in the hard hit area of Lombardia, hence not everyone in need of ICU hospitalization had access to it. The second problem is that the daily numbers given by Italian Civil Protection only mention the total number of people hospitalized in ICUs in that day and not the daily incremental number. It is not possible, therefore, to know the real cumulative number of people hosted in ICUs, because we do not know how many people each day left them due to recovery or by death. 

The only number that has rigorous statistical meaning, then, is the daily cumulative number of deaths. We choose to use this number in order to statistically analyse the evolution of the epidemic and to predict its end. Obviously, since we are interested in determining the time at which the epidemic will end and the total number of infected people at the end of the epidemic, we have to correctly consider the relation of the daily cumulative number of deaths with the cumulative number of infected people. The number of deaths is linked to the number of infections by the IFR = D/I (D = deceased, I = infected); then, correcting the number of deaths for the constant factor represented by the inverse of IFR (I/D) gives the number of infected people. However, we must also consider that infection and death are two temporal limits of the disease; it begins with infection, manifests symptoms, then ends with one of two possibilities: recovery or death. To account for this, we must consider the shift in time between infection and death. For COVID-19, we estimate that the average time from infection to death in Italy is 16 days. This shift is computed as the sum of the median incubation time, estimated to be comprised between 4.8 and 7.1 days [26,27,28], and the median time from symptoms onset to death, that is 10 days in Italy according to ISS [29]. A similar time has also been observed in China when considering only patients over 70 years old [30] consistent with the median age of people who died in Italy due to the infection being 79 years [29]. With such relations in mind between infections and deaths, we fit the cumulative number of deaths to a logistic function of the general form: $y\left(t\right)=K\frac{1+me^{-tr}}{1+qe^{-tr}}$. 

**Figure 2:**
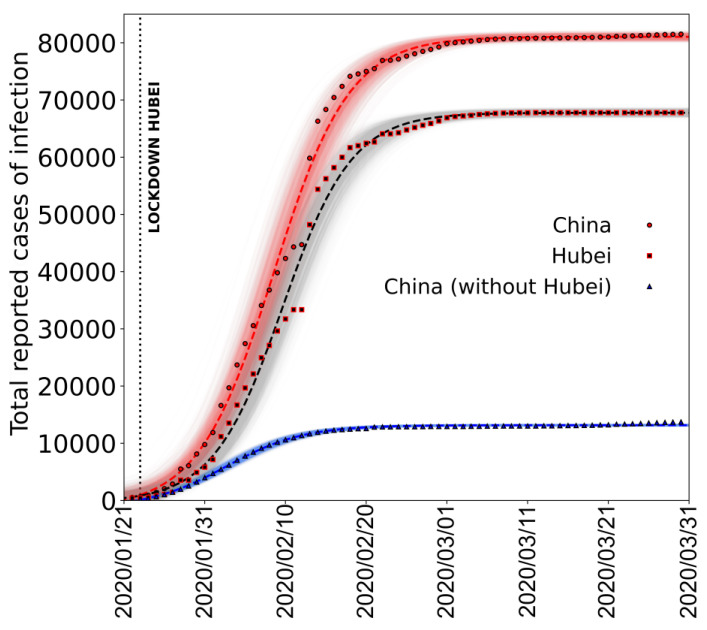
Total COVID-19 cases reported in China from 22 January to 31 March 2020 according to the Johns Hopkins University data repository. Circles, squares, and triangles represent total COVID-19 cases registered in China, the region of Hubei, and China without Hubei; red, black, and blue dashed lines are the associated logistic fits. Shaded areas represent the family of curves obtainable by making the fit parameters vary within their confidence intervals. Fit parameters: Logistic $(y=K\frac{1+me^{-xr}}{1+ne^{-xr}};China:K\;=\left(811\pm 3\right)\cdot 10^{2},\;m=-0.9\pm 0.6,\;n=53\pm 9,\;r=0.214\pm 0.008);$ $Hubei:\;K=\left(678\pm 3\right)\cdot 10^{2},\;m=-0.4\pm 1.2,\;n=100\pm 20,\;r=0.232\pm 0.01;$ $China\;wihtout\;Hubei:\;K=\left(131.6\pm 0.3\right)\cdot 10^{3},\;m=-1.4\pm 0.11,\;n=14\pm 1.4,r=0.208\pm 0.006).$

To validate the use of this function, in Figure 2, we show the fit made using a logistic function, to the epidemic curve of the cumulative daily number of cases (infections) reported in the whole China, Hubei province and China without the Hubei province [31].

It is clear that, after the initial epidemic spread is over, the overall epidemic behaviour from the onset to end of infection can be well described by the logistic function. 

**Figure 3:**
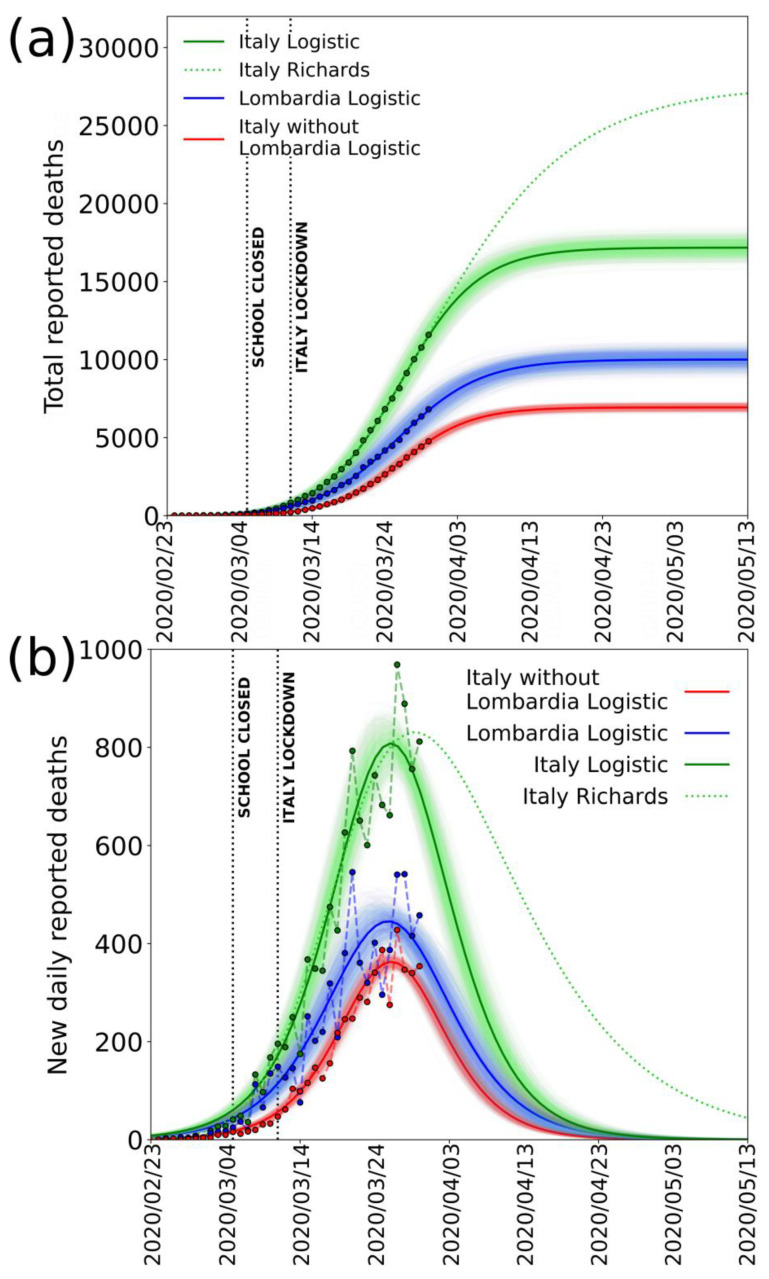
Deaths reported by Italian Civil Protection. (**a**) Total deaths reported in Italy (green dots), Lombardia (blue dots), and Italy without Lombardia (red dots) from 24 February to 30 March 2020 and the corresponding logistic fit in solid lines. Dotted vertical lines mark the dates of Italian school lockdown and Italy total lockdown. A sample best fitting Richards’ curve for the whole Italy is also shown (green dotted line). (**b**) Same as in (**a**) using the new daily reported deaths and fitting the derivate of the logistic and Richards’ curve. Shaded areas represent the family of curves obtainable by making the fit parameters vary within their confidence intervals. Fit parameters: Logistic $(y=K\frac{1+me^{-xr}}{1+ne^{-xr}};Logistic^{\prime }:\;\frac{Kr\left(m-n\right)e^{-xr}}{{\left(e^{rx}+n\right)}^{2}};Italy:\;K=\left(178\pm 4\right)\cdot 10^{2},\;m=-3.1\pm 0.5,n=380\pm 30,\;r=0.183\pm 0.004);$ *Lombardia*: $K=\left(100\pm 4\right)\cdot 10^{2},\;m=-2.8\pm 0.5,\;n=270\pm 30,r=0.176\pm 0.006);$ *Italy without Lombardia*: $K=\left(74\pm 2\right)\cdot 10^{2},\;m=-4.0\pm 0.8,\;n=740\pm 60,r=0.201\pm 0.004;$ $\mathrm{Richards}(y=\frac{K}{{\left(1+e^{-B\left(x-t\right)}\right)}^{\frac{1}{\nu }}};y^{\prime }:\frac{BKe^{\left(t-x\right)}{\left(e^{\left(t-x\right)}+1\right)}^{\frac{-\left(\nu +1\right)}{\nu }}}{\nu };Italy:\;K=\left(276\pm 5\right)\cdot 10^{2},$  B=0.1±0.5,t=20±30, ν=0.196±0.004).

To describe the evolution of the epidemic in Italy, as we explained, we decided to use the cumulative daily number of deaths to fit the logistic function because it is a much more reliable quantity when compared to the total reported cases. Figure 3a,b shows the fits of the data for deaths reported in Italy (green), Lombardia (blue), and Italy without Lombardia (red) until 30 March 2020, with a logistic function and its derivative, respectively. From the derivative of the logistic function, we observe that the model gives a fairly good fit to the data until 26 March 2020. After this date, the number of deaths remains high and perturbs the data near the peak and does not decrease as the curve would previously predict. If we separate the data of Lombardia from the rest of Italy, however, we see that the result is much more regular (red line, Figure 3b). 

**Figure 4:**
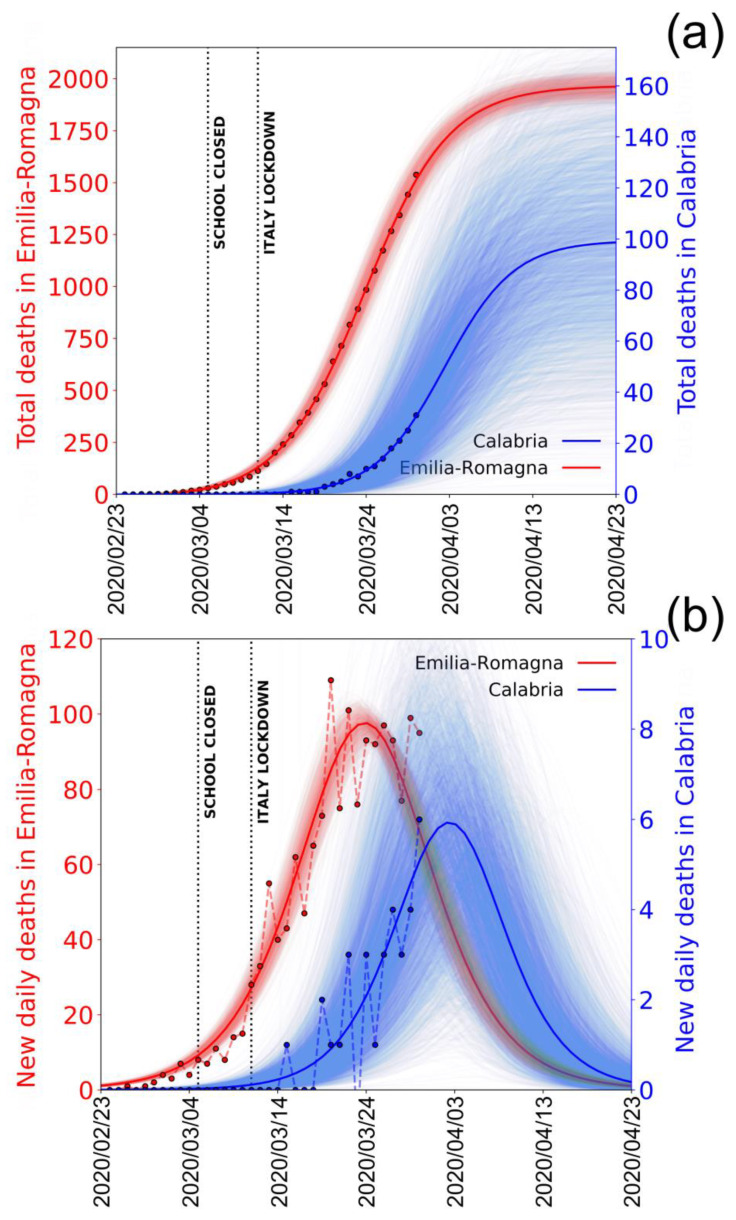
Deaths reported by Italian Civil Protection for Emilia-Romagna and Calabria regions. (**a**) Total, cumulative deaths reported in Emilia-Romagna (red dots) and in Calabria (blue dots) from 24 February to 30 March 2020 according to Italian Civil Protection and the corresponding logistic fit obtained from the data. Dotted vertical lines mark the dates of Italian school lockdown and Italy total lockdown. (**b**) Same as in (**a**) using the new daily reported deaths and fitting the derivate of the logistic curve. Shaded areas represent the family of curves obtainable by making the fit parameters vary within their confidence intervals. Note: the left and the right y-axes scales refer to the Emilia-Romagna and the Calabria curves, respectively. Fit parameters: Logistic $(y=K\frac{1+me^{-xr}}{1+ne^{-xr}};Logistic^{\prime }:\;\frac{Kr\left(m-n\right)e^{-xr}}{{\left(e^{rx}+n\right)}^{2}};;Emilia-Romagna:\;K=1970\pm 40,\;m=-3.0\pm 0.6,n=360\pm 30,\;r=0.197\pm 0.004);$ $\;Calabria:\;K=100\pm 20,\;m=-30\pm 13,\;n=\left(12\pm 3\right)\cdot 10^{3},r=0.208\pm 0.006).$

The curves, the logistic, and the derivative are both well constrained in this case, and the peak value seems to have occurred on 27 March. The anomaly of the data for Lombardia is even more evident by considering the data of Emilia Romagna Region, which is the second most affected region according to the official reports [8]. Figure 4a,b shows in red the logistic fit (and its derivative) to the cumulative (and the new) number of deaths in Emila Romagna. 

In this case, too, it is evident the data are well fitted and the curves well constrained. The peak value occurs around 24 March 2020, consistent with this region being among the first where the infection began. Besides Emilia-Romagna, which represents a good example of a region in which the epidemic is already “mature” (in the sense that the peak of deaths has already passed), it is interesting to see what happens in one of the southern regions of Italy, for example, Calabria, where the infection presumibly started later. These data are also shown in blue in Figure 4 for comparison. It is clear that, since the epidemic here has not yet reached the peak value, the curves are poorly constrained, and the future evolution is still very uncertain. The difference between Emilia-Romagna and Calabria curves illustrates well the different behaviours of the epidemic in the different regions and the difference between northern and southern regions.

To estimate the cumulative number of infections, we use several tentative values of IFR: the one computed from the University of Oxford, IFR = 0.2%; the one computed from the lethality on the Diamond Princess, IFR = 1.3%; and one much larger, IFR = 5.7%, in case the IFR for Italy would be much larger than in other countries due, for instance, to a rapid hospital and ICU saturation and/or high median age of people who died due to the infection (79 years) [29]. This IFR value was obtained considering the actual CFR computed by Italian authorities (11.3%) and taking into account the percentage of undiagnosed asymptomatic cases, which is found up to 50% by recent studies [9,12,26]. Such a large range of IFRs for conversion is useful to check the final number of total infections but also to determine the portion of unreported cases in respect to the official numbers.

**Figure 5:**
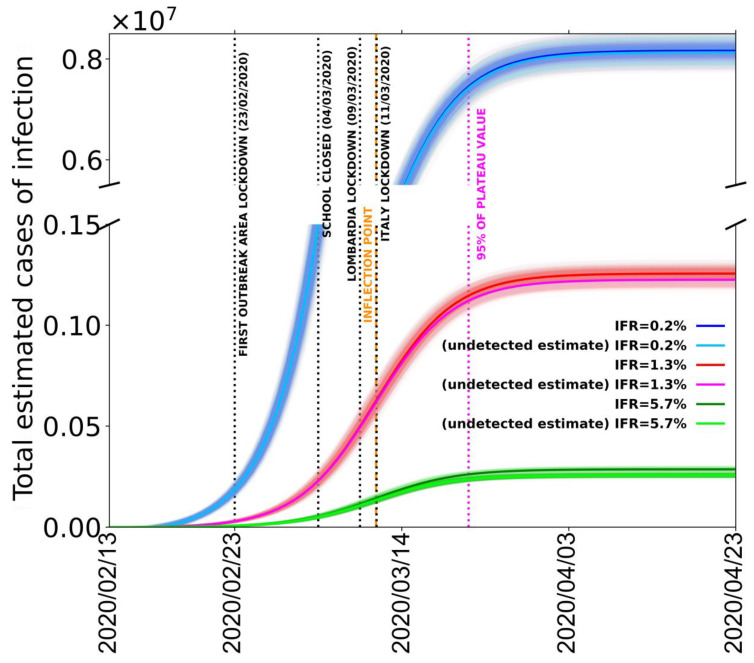
Estimated (total and undetected) COVID-19 cases in Italy based on three different infection fatality ratio (IFR) hypotheses: 0.2% (blue and light blue lines), 1.3% (red and pink lines), and 5.7% (green and light green lines). Blue and sky-blue solid lines represent logistic fits of total and undetected estimated cases with IFR = 0.2%, respectively. Red and pink solid lines represent logistic fits of total and undetected estimated cases with IFR = 1.3%, respectively. Green and light green solid lines represent logistic fits of total and undetected estimated cases with IFR = 5.7%, respectively. Black dotted vertical lines mark the dates of Codogno area lockdown, Italian schools’ lockdown, Lombardia lockdown, and Italy lockdown. Dark orange dashed vertical line marks the inflection points of the three curves representing the total infected estimates; magenta dotted vertical line marks the 95% of the plateau of the three curves. Shaded areas represent the family of curves obtainable by making the fit parameters vary within their confidence intervals. Best fit parameters are listed in Table 2.

In order to estimate the total number of infected cases, we take the less-noisy data from Italy without Lombardia, but we normalize the number of deaths to be equal to the total Italian deaths (including the Lombardia data) at the last date (30 March 2020). This procedure is used in order to remove the data scattering introduced by the Lombardia reported number of deaths without, on the other hand, underestimating the total number of infections. In Figure 5, we report the fit of the estimated amount of total cases in all of Italy with the procedure that we just described. 

The different logistic curves (blue, red, and green) present in the figure are the estimated number of contagious people for the different IFR values of 0.2%, 1.3%, and 5.7%, respectively. From these values, we also subtract the number of cases reported by Italian Civil Protection to estimate the number of unreported contagious cases (light blue, pink, light green); the parameters obtained from the best fits are reported in Table 2.

It is evident that the number of reported infection cases is only a minor fraction of the estimated number of contagious people. In particular, for an IFR of 0.2%, 1.3%, and 5.7%, we estimate in the whole of Italy a total number of cases at 30 March 2020 (35 days from the first declared red zone) of about 8 million, 1.2 million, and 300,000, depending on the 0.2, the 1.3, and the 5.7% IFR values, respectively. With a total number of reported cases of about 100,000, Italy might be strongly underestimating the total number of infected people (including asymptomatic and pauci-symptomatic persons) of 98.7%, 91.7%, or 63.7% depending on the IFR. From the time dependence of the logistic function, we observe that the inflections of the respective curves are now exceeded by the data points no matter which conversion factor IFR is used. This probably means that Italy as a whole has already overcome the maximum number of new daily infections. In particular, the peak value of infections should have been reached around 11 March 2020, one week after the closing of schools and just after the lockdown of Italy. Moreover, our results predict that the 95% point of the maximum value of the best fitting logistic curves was reached in the last week of March. By the first week of April, the real number of contagious people was already well within the saturation thus ostensibly should not increase anymore. Finally, by tracking the dates when the logistic functions of the estimated number of contagious people have value 1, we can also obtain an estimate for the beginning of the epidemic in Italy. Depending on the used IFR, we find a starting date between 23 January and 30 January 2020, confirming the epidemiological data survey completed in Lombardia [5].

It is possible that, after the epidemic spread in Italy is over, growth functions different from the logistic such as Gompertz [32], Janoschek [33] or Richards [34] sigmoids could be more suited to describe the epidemic spread because they allow their derivative to be non-symmetric and therefore can result in a more generalized integral function. A non-symmetric integral curve with a slower derivative in the descending part after the peak could result, for istance, from a differential shift of epidemic curves for different regions—particularly for the southern ones—due to the delay of the infection start in Lombardia. When compared to the logistic function, a typical variation of these more generalized growth functions is the shift of the inflection point a few days later and the increase of the saturation value. Because here we are highlighting an extreme underestimation of the reported cases in Italy already with a simple logistic function, our main result would be even more inflated by the use of other growth functions. However, for additional clarity, we reported in Figure 3 (green dotted curve) an example of Richards curve, giving a reasonably good fit to whole Italy data until 30 March. It is important to note that, with these data not yet describing the decay after the peak, the Richards curve is very poorly constrained. It is evident nevertheless that, using such a curve to estimate the total number of infections, due to a much lower decay, the saturation would occur many days after, and the final, cumulative number of deaths (and of infected) would be much larger (more than 50% with this particular curve).

## 6. Discussion

The COVID-19 epidemic in Italy shows very abnormal features in terms of the severity of disease and its lethality rate. The CFR (defined as the ratio between the number of deceased and the number of recorded infected) in Italy reached very high values disproportional to those of other countries. This CFR reached approximately 11% in the country overall with a rate of 16% in the Lombardia region, which represents half of the total recorded infections in Italy. Estimating a reliable value of the IFR (the ratio between the number of deaths and the total number of infections) during the epidemic is very difficult due to the complex laboratory procedures required to diagnose it. In this case, we considered three approaches: the first one, used by University of Oxford [2], is based on the minimum reliable CFR values and assuming a 50% rate of non-recorded infections; the second one uses the unusual laboratory-like case study provided by the Diamond Princess cruise ship on which all passengers and crew (3771 people) were tested for SARS-CoV-2; a third one using the CFR computed by Italian authorities and assuming, as in the first case, that the detections have lost almost all asymptomatic and pauci-symptomatic individuals. University of Oxford computed a value of IFR = 0.2%. In the second approach, we considered that, on the Diamond Princess cruise ship, 712 infections were detected and 9 of them died. This permits a mostly unbiased value of IFR = 1.3% to be calculated. In the third case, from a CFR = 11.3%, we computed an IFR = 5.7%. We then consider these values as the limits defining the range of IFR. If we assume that the infection number underestimation is the only reason for the large overestimation of the lethality rate, to determine the real number of infected cases and correct the apparent lethality to the true value, we should multiply the “official” number of recorded infections by the ratio between the CFR = 11.3% and the IFR (=0.2%, 1.3%, or 5.7%). In this way, we obtain a real number of infections in Italy (as of 30 March 2020) that ranges between about 0.3 and 8 million people infected (Figure 5).

While this number appears very high in respect to the total number of reported cases of less than 90,000 in China (where, using the same assumption and the central IFR value, this amount should be multiplied by four), it is not unreasonable for several reasons. While a high number of tests have been completed in Italy (about 500,000), this is still only a small fraction of the total population (60 million people) and does not take into account the number of asymptomatic and pauci-symptomatic cases. This is also confirmed by observing that the CFR in Italy is very similar to the percentage of deaths detected in hospital cases [11]. The large number of potential daily infections in Italy during commutes to work or study can be also independently estimated by the statistics for the use of public transportation (the most likely to cause infection due to the high number of people assembled in small space). Before the epidemic spread and the lockdown measures, roughly 30 million people used to travel in Italy each day for work or study, with peak values in Lombardia [35]. Roughly 56% of these people use public transportation; two thirds of the total travel for more than 15 min. Considering only these people and not including gatherings of people in schools, work places, and occasional visits to restaurants, clubs, pubs, sport matches, theatres, supermarkets, etc., we get a minimum estimate of about 11 million people within close contact to one another each day. A final indication that the number of effective infected cases is not unrealistic comes from the exponential fit of the first days of infection (Figure 1); by extrapolating that exponential curve to 30 March 2020, it would predict about one million infection cases. Obviously, such a hypothesis would assume that the exponential increase lasted until 30 March 2020 (or until a few days before) and that the real exponential curve was missed because the limited number of tests progressively sampled a smaller and smaller percentage of the true daily number. From any point of view, in conclusion, a real number of 0.3–8 million currently infected people does not appear unrealistic.

Other factors could also affect the very high lethality observed (as CFR); discarding the existence in Italy of a virus strain significantly different and more aggressive (a hypothesis impossible to verify because there is not yet the Italian genome available), a possible contribution could come from the relatively high air pollution (from particulate matter PM10, PM2.5, and ozone) of the area around the Po Valley, where more than 50% of counted infections are clustered. Among the other hypothesized causes, the high average age (however lower than Japan, which showed a much lower CFR) is not likely to play a fundamental role, nor is the number of smokers (lower than the EU average). The observed high antibiotic-resistance, the highest in Europe, has been also indicated as a possible contributing factor. This resistance results in the deaths in Italy of roughly one third (10,000 cases; double that of France and four times that of Germany) of the total deaths from antibiotic resistance in the entire EU (33,000 cases), yet it does not appear to significantly affect the lethality (CFR) associated with seasonal flu (the most reliable comparison we can do to infer the possible effect on COVID-19 lethality), which seems not significantly higher with respect to other European countries. 

Another issue that often comes out as a possible bias in the lethality estimates for Italy is the way of counting equally people dead “with” or “by” the infection. We, however, do not think this question can seriously affect the data provided that only those who die for pulmonary collapse (or else for collapse of other organs following a serious pulmonary disease) are counted. On the contrary, an interesting issue given the high selectivity of the disease towards older and/or multi-pathology people is to understand, at the end of the year, the total deaths caused cumulatively by all the seasonal diseases (flu, infections from antibiotic-resistant bacteria, walking pneumonia, COVID-19).

A significant contribution to the increase of the “local” IFR could, on the contrary, have come from the saturation of the public hospitals, in particular from the limited number (as compared, for instance, to Germany) of ICU beds. Italy before the COVID-19 epidemic had 8.4 ICU beds per 10,000 citizens (5090 ICU total), whereas Germany had 34 of them (28,000 ICU total). Faulty actions taken by the public hospitals in managing the first days of infection (testified by many media and by a dramatic percentage of infected medical staff) could have significantly enhanced the infection. As of 30 March 2020, 51 doctors had died and 6414 medical staff members have been infected, most of them in Lombardia (ISSb, 25 March 2020). The anomalous behaviour of a persistently high number of deaths in Lombardia also suggests some problems in that region, which has the most critical numbers and where the infection started. Such problems likely include an insufficient sanitary system that was stressed above the critical point by the extreme numbers of people hospitalized (11,883) or in ICUs (1324). One of the main criticisms in Lombardia, largely reported by media and also focused upon by some judicial inquiries, has been the transfer of COVID-19 patients in the RCH (Residential Care Homes, the long stay hospitals for old people, the terminally ill, and people with disabilities), which caused a large number of deaths [29,36].

In this paper, we also use an indirect procedure based on the analysis of the cumulative number of deaths—the only reliable datum—to forecast the short-term evolution of the epidemic in Italy. As we discussed, the number of recorded infections is not statistically reliable for such analyses, as they are inhomogeneous in time and in space among different regions. Considering the average time span from infection date to death (16 days), we show that lethality data converted to infection data using a large range of possible IFR are well fitted by logistic functions; this indicates that the 95% rate of total infection (i.e., the starting of the flattening of the function, which represents the end of infection) is expected near the last days of March. The point of inflection of the logistic best fitting functions, which corresponds to the peak of the infections in Italy, occurred around 11 March 2020 given the interval of confidence over the delay time from symptoms to death. Looking at Figure 5, this date occurs just after the national lockdown of the Italy, and it demonstrates the successful mitigating impact of the lockdowns; however, since it also occurs only from 4 to 10 days after the closing of the schools, it probably indicates that this measure has also been effective in containing the infection. Indeed, the high effectiveness of the closure of schools for containing epidemic spread has been specifically assessed by a number of epidemiologic studies (i.e., [37]).

Our results also illustrate the shift of the infection curves between the northern regions where the infection started (i.e., Emilia-Romagna) and the southern Italian regions (i.e., Calabria) where, on 30 March 2020, the peak had not been reached yet and therefore the curves are still unconstrained and the future trends very uncertain. These results, based on the rate of casualties, further confirm that the infection data we record are dependent on variable and inhomogeneous sampling and not the true statistical evolution of the epidemic. Looking at these very inconclusive data, it is clear that the increase or the decrease of new daily infections strongly depend on the number of tests performed. This is an obvious consequence of the fact that the true number of infections is much larger than the small sample tested. The more tests you do, the higher the number of cases you record. The conclusion that the epidemic in Italy is reaching saturation and is predicted to end within the first half of April, although obscured by the tested number of new infections, should be seriously considered in order to determine how and when to commence the next stages of the infection containment response.

## 7. Conclusions

In this paper, we analysed the COVID-19 epidemic in Italy, which was amongst the largest and the most lethal in the world. We discussed the causes of such anomalous behaviour of the disease in Italy, where lethality appears much higher than in other countries. Among the various hypotheses made to explain such anomalous behaviour, it appears that neither the older median age of the population nor the observed antibiotic-resistance of the population (by far the highest levels in Europe), nor even the smoking rate should have significant effects on the observed lethality. The most reasonable effect would involve a strong underestimation of the extension of the infection (implying a factor 2.8 to 56.5 more cases than the tested ones). A possible further contribution could be given by the very high fine powder and ozone pollution in the Po Valley (one of the highest ones in Europe). This could facilitate the virus transmission as well as make the lungs more vulnerable and stressed, causing heavier damages upon impact with the virus. Another factor emerging from our analyses (as well as from media) that likely amplified lethality could have been the lack of preparedness and initial faults of the sanitization protocols in hospitals, particularly in Lombardia, where the epidemic first emerged.

The problem of underestimation of the number of infections coupled with inconsistent sampling and testing of the positive cases makes these data unreliable to use for statistics aimed to forecast the epidemic evolution. Here, we use the cumulative daily number of deaths corrected for an appropriate IFR to simulate the evolution of the epidemic by a logistic function. When corrected for IFR and time shift between infection and death, these data can be well fitted by a logistic function and show that the epidemic started at the end of January, the actual peak of infection was reached around mid-March and the saturation of the curve (end of epidemic) is expected within the first week of April or a few days later. For the southern Italian regions, the saturation of the curve could be possibly delayed by a few days. The information presented here regarding the possible evolution of the epidemic and its likely end, which is not evident from the very incomplete sampling of the population, should be seriously considered in the decision-making process per measures to relax lockdown in the future. Equally, it is evident that a mass test campaign (for infection and for antibodies) is required to correctly understand and control the evolution of the epidemic. The apparent effectiveness of school closures, probably the least invasive measure in a social and economic sense, should be further investigated by the government and considered as a primary measure. The study of this unprecedented medical catastrophe will hopefully give robust indications to avoid, in the future, the same errors. It is also requisite to improve the health system both in hospital and in primary health care settings, as they are invaluable to a functional society. 

## Figures and Tables

**Table 1:** Numbers of recorded infections and deaths in several countries as of 30 March 2020. Also indicated is the CFR (case fatality ratio) defined as the ratio between the number of deaths and the number of recorded cases.

Country	Cases	Deaths	CFR (%)
Lombardia (Italy)	42,161	6818	16.1
Italy	101,739	11,591	11.3
Spain	87,956	7716	8.77
Netherlands	11,750	864	7.35
France	44,550	3024	6.78
Iran	41,495	2757	6.64
UK	22,141	1408	6.35
Belgium	11,899	513	4.31
China	81,518	3305	4.05
Sweden	4028	146	3.62
Denmark	2577	77	3.53
Japan	2178	57	2.61
Switzerland	15,922	359	2.25
USA	163,788	3141	1.91
Ireland	2910	54	1.80
South Korea	9661	158	1.63
Turkey	10,827	168	1.55
Canada	7448	89	1.19
Austria	9618	108	1.12
Portugal	6408	140	1.11
Germany	66,885	645	0.96
Norway	4445	32	0.71
Australia	4460	19	0.42
Israel	4695	16	0.34

**Table 2:** Logistic fit $\left(y=K\frac{1+me^{-xr}}{1+ne^{-xr}}\right)$ parameters for the estimated (total and undetected) COVID-19 cases in Italy based on three different IFR hypotheses: 0.2% (blue and light blue dots), 1.3% (red and pink dots), and 5.7% (green and light green dots) (see Figure 5).

**Total Estimated Cases (IFR 0.2 %)**			
K: (81 ± 1.6)∙10^5^	m: −3.7 ± 0.7	q : 860 ± 60	r : 0.211 ± 0.003
**Total Undetected Cases (IFR 0.2 %)**			
K: (80 ± 1.6)∙10^5^	m: −3.7 ± 0.7	q : 860 ± 60	r : 0.211 ± 0.003
**Total Estimated Cases (IFR 1.3 %)**			
K: (137 ± 3)∙10^4^	m: −3.1 ± 0.5	n : 380 ± 30	r: 0.184 ± 0.004
**Total Undetected Cases (IFR 1.3 %)**			
K: (132 ± 3)∙10^4^	m: −3.1 ± 0.5	n: 380 ± 30	r: 0.183 ± 0.004
**Total Estimated Cases (IFR 5.7 %)**			
K: (290 ± 9) ∙10^3^	m: −3.6 ± 0.6	n: 890 ± 50	r: 0.213 ± 0.003
**Total Undetected Cases (IFR 5.7 %)**			
K: (260 ± 6) ∙10^3^	m: −3.4 ± 0.6	n: 860 ± 50	r: 0.215 ± 0.003
